# Antimicrobial Drug–Resistant *Escherichia coli* in Wild Birds and Free-range Poultry, Bangladesh

**DOI:** 10.3201/eid1812.120513

**Published:** 2012-12

**Authors:** Badrul Hasan, Linus Sandegren, Åsa Melhus, Mirva Drobni, Jorge Hernandez, Jonas Waldenström, Munirul Alam, Björn Olsen

**Affiliations:** Author affiliations: Uppsala University Department of Medical SciencesUppsala, Sweden (B. Hasan, Å. Melhus, M. Drobni, B. Olsen);; Linnaeus University Centre for Ecology and Epidemiology in Microbial Model Systems, Kalmar, Sweden (B. Hasan, J. Hernandez, J. Waldenström, B. Olsen);; International Center for Diarrhoeal Disease Research, Bangladesh, Dhaka, Bangladesh (M. Alam);; Uppsala University Department of Medical Biochemistry and Microbiology, Uppsala (L. Sandegren).

**Keywords:** Escherichia coli, extended-spectrum β-lactamase, ESBL, CTX-M gene, antibiotic, antimicrobial resistance, bacteria, wild birds, poultry, sequence type, Bangladesh

## Abstract

Multidrug resistance was found in 22.7% of *Escherichia coli* isolates from bird samples in Bangladesh; 30% produced extended-spectrum β-lactamases, including clones of CTX-M genes among wild and domestic birds. Unrestricted use of antimicrobial drugs in feed for domestic birds and the spread of resistance genes to the large bird reservoir in Bangladesh are growing problems.

Dissemination of *Enterobacteriaceae* that produce extended-spectrum β-lactamases (ESBLs) is increasing in humans and animals globally (1,2). Clinically relevant sequence and ESBL types have been reported among wild birds (3). *Escherichia coli* strains from domestic animals and poultry tend to carry the same CTX-M enzyme variants that are locally dominant in human isolates (4). Using birds as sentinels of the spread of antimicrobial drug resistance in the environment could indicate a wider prevalence of drug-resistant disease in humans (3,5).

In Bangladesh, the problem of antimicrobial drug resistance in humans and poultry is augmented by the uncontrolled use of unprescribed antimicrobial drugs (*6*). A high prevalence of resistant phenotypes has recently been reported in poultry and human *E. coli* isolates from Bangladesh (*6*,*7*). ESBL-producing *E. coli* and *Klebsiella pneumoniae* are common in clinical settings (*8*), but data quantifying the prevalence of different ESBL genotypes are limited. We screened fecal samples from wild birds and from poultry in the Rajshahi district of Bangladesh for antimicrobial-resistant and ESBL-producing *E. coli*.

## The Study

Samples from 96 birds (41 wild ducks, 29 chickens, 23 ducks, and 3 geese) were collected from the Padmachar area of Rajshahi District during January 2009. In this area, a lake hosts several thousand wintering wild birds; that lake also is frequented by poultry from surrounding households. Each fecal sample, collected by swirling a cotton swab in a bird’s cloaca or droppings, was submerged in a bacterial freeze medium and handled as described (*5*). Each sample was placed on an Uriselect 4 agar plate (Bio-Rad Laboratories, Marnes-La-Coquette, France), and assessed for *E. coli* by biochemical testing and API 20E biochemical strips (bioMérieux SA, Marcy-l'Etoile, France). One *E. coli* isolate per positive bird sample was tested by disk diffusion against 15 antimicrobial drugs (Table 1) according to the recommendations of the European Committee on Antimicrobial Susceptibility Testing (www.eucast.org). Multidrug resistance was defined as resistance to at least 3 classes of antimicrobial drugs.

**Table 1:** Antimicrobial drug resistance phenotypes in randomly isolated *Escherichia coli* from wild and domestic birds, Bangladesh

Drug	Domestic birds, n = 52	Wild birds, n = 14
Tetracycline	24	1
Trimethoprim/sulfamethoxazole	17	2
Ampicillin	15	4
Cephradine	0	1
Cefuroxime	2	1
Cefadroxil	0	1
Nalidixic acid	11	1
Ciprofloxacin	3	1
Streptomycin	3	1
Gentamicin	0	1
Fosfomycin	0	1
Chloramphenicol	4	0
Nitrofurantoin	1	0
Tigecycline	0	0
Mecillinam	0	0

Each sample was also enriched in brain-heart infusion broth (Becton Dickinson, Franklin Lakes, NJ, USA) supplemented with vancomycin 16 μg/mL (ICN Biomedicals Inc. Aurora, OH, USA) for 18 h at 37°C. For detection of ESBL-producing bacteria and genes (*bla*_CTX-M_, *bla*_SHV_, and *bla*_TEM_) in putative ESBL isolates, described methods were used (*5*,*9*). Carbapenem-resistant isolates were screened on Mueller-Hinton agar plates supplemented with 2 µg/mL or 8 µg/mL meropenem and incubated overnight at 37°C.

The genetic profiles of the ESBL-producing *E. coli* isolates were determined by using repetitive element PCR. The reaction mixture contained 1× Taq PCR buffer, 0.625 µmol/L primer ERIC1R (5′-ATGTAAGCTCCTGGGGATTCAC-3′), 1.9 mmol/L MgCl_2_, 50 µmol/L dNTPs, 0.6 U Taq polymerase, and template in a total volume of 20 μL. Cycling parameters were 1 min at 94°C; 1 min at 36°C and 2 min at 72°C for 45 cycles, and a final extension for 5 min at at 72°C. Isolates that had identical strong band patterns but an addition or a loss of a weak band were assigned subtype numbers.

One representative for each repetitive element PCR genotype and subtype (n = 18) was characterized by multilocus sequence typing (MLST) (10). After sequencing, allele profiles and sequence types were determined by using the *E. coli* MLST database (http://mlst.ucc.ie/mlst/dbs/Ecoli/#). One representative sample for each genotype was tested for transferability of the ESBL plasmid by conjugation to recipient *E. coli* DA11782 (*mcrA, Δmrr-hsdRMS-mcrBC*, *ΔlacX74, deoR, recA1, araD139Δ* [*ara-leu*] *7697, galK, rpsL, endA1, nupG*, *rif^R^*). Equal amounts of donor and recipient overnight cultures in Luria-Bertani broth were mixed and incubated, without shaking, overnight at 37°C. Approximately 10^9^ CFU of conjugation mixture was placed on selective plates containing 10 µg/mL cefotaxime, 100 µg/mL rifampin, and 50 µg/mL nalidixic acid and incubated overnight at 37°C.

*E. coli* was isolated from 66 samples, yielding an isolation rate of 73.3% regardless of bird species. Thirty-five (53%) of the 66 isolates were resistant to ≥1 antimicrobial compounds. The most common resistance was to tetracycline. The 3 next most common resistances were to ampicillin, trimethoprim/sulfamethoxazole, and nalidixic acid (Table 1). Multidrug resistance was found in 22.7% (15/66) of the isolates, and 13.6% (9/66) of the isolates were resistant to 4 or 5 classes of antimicrobial drugs. Screening for carbapenamase producers yielded no isolates.

The overall prevalence of ESBL carriage among birds was 30% (27/90); 36 *E. coli* isolates produced ESBL. Thirty-four of them belonged to the CTX-M-1 group (2 *bla*_CTX-M-1_ and 32 *bla*_CTX-M-15_) and 2 to the CTX-M-9 group, the latter of which were CTX-M-14–like. Combinations of *bla*_CTX-M-15_ or *bla*_CTX-M-1_ and *bla*_TEM-1_ were detected in 50% of the isolates, whereas none harbored SHV-genes.

The genetic fingerprints of the ESBL-producing *E. coli* isolates identified 15 genotypes, of which 19 (53%) of 36 were type A (Table 2).This genotype was found in wild and domestic birds. MLST analysis revealed 15 different sequence types (STs) and 1 nontypeable isolate (Table 2). Four isolates had new allele types or a new combination of allele types and were given novel STs (ST2690–ST2693). STs found in wild birds differed from those in poultry. One CTX-M-14–producing isolate from chicken belonged to the internationally recognized ST131 clone. Conjugation was successful for 9/18 isolates, indicating the transferability of plasmids carrying ESBL genes.

**Table 2:** Antimicrobial drug resistance genotype classification of ESBL-producing *Escherichia coli* isolates from wild birds and free-range poultry, Bangladesh*

Host	Isolate	ESB type	Repetitive element PCR genotype	Sequence type
Common teal (*Anas crecca)*	B51	CTX-M-15	A4	ST1408
Common teal	B53	CTX-M-15	A	ST1408
Tufted duck (*Aythya fuligula)*	B66	CTX-M-15	A3	ST1312
Domestic duck *(Anas platyrhynchos)*	B93	CTX-M-15	E	ST2141
Domestic duck	B97	CTX-M-15	G	ST2690†
Domestic duck	B98	CTX-M-15	F	ST448‡
Domestic duck	B100	CTX-M-15	D	ST405§
Domestic duck	B102	CTX-M-15	H	ST2691†
Domestic duck	B106	CTX-M-15	I	ST648
Domestic chicken *(Gallus domesticus)*	B125	CTX-M-15	A2	ST206¶
Domestic chicken	B127	CTX-M-1	J	ST744
Domestic chicken	B129	CTX-M-15	K	ST648
Domestic chicken	B130w	CTX-M-14 like	L	ST131
Domestic chicken	B133	CTX-M-15	M	ST2450
Domestic chicken	B136P	CTX-M-15	N	ST2692†
Domestic chicken	B137P	CTX-M-1	O	ST744
Domestic chicken	B140	CTX-M-15	A	ST2693†
Domestic chicken	B143	CTX-M-15	B	ST224

*ESBL, extended-spectrum β-lactamase. †New sequence type. ‡Clonal complex ST448. §Clonal complex ST405. ¶Clonal complex ST206.

## Conclusions

The carriage rate of ESBLs was high and the predominating antimicrobial-resistant phenotypes of wild birds and poultry appeared to correlate with antimicrobial prescription patterns in Bangladesh (*6*). Most ESBL-positive samples originated from poultry, and household poultry was the predominant carrier of the *bla*_CTX-M-15_ genotype and the CTX-M-14–like enzymes. However, the *bla*_CTX-M-15_ genotype was retrieved from wild birds. The CTX-M-15 gene shows a global distribution in clinical settings but has been reported from poultry in the United Kingdom (*11*) and from wild birds in Sweden (*5*), which indicates that this ESBL type also is widely disseminated in the environment.

The PCR-based genotyping showed the diversity of the ESBL-producing *E. coli* isolates. Wild birds and domestic poultry harbored the same strains, and some of the ducks had the same strains as chickens. This commonality of strains might be caused by a common use of natural water resources, and shows with what ease *E. coli* can travel between species.

MLST analysis identified several human-associated genotypes, including ST448, ST405, ST744, ST648, and ST131. The epidemic *E. coli* strain O25bST131 did not carry the more common CTX-M-15 gene but a CTX-M-14–like gene, a frequent finding in hospitals in Taiwan (*12*). Metallo-β-lactamases of the New Delhi metallo-β-lactamase type have not been found in the environment of Bangladesh (*13*), but ST405 and ST648 are associated with New Delhi metallo-β-lactamase-1–producing organisms on the subcontinent of India (*14*). Finally, *E. coli* ST744 carried in this study CTX-M-1. ESBL-producing *E. coli* ST744 has been reported previously in humans in Laos (*15*).

We showed that *E. coli* that produces CTX-M-15 is endemic to birds in Bangladesh. Our findings suggest that wild birds and free-range poultry might be crucial environmental indicators of antimicrobial drug resistance. They also might take a more active part than previously thought as spreaders and as long-term reservoirs of medically threatening pathogens and resistance genes. Several factors are likely to contribute to the widespread dispersal of ESBLs in Bangladesh, including dense population, poor sanitation, and close contact with livestock combined with a high selective pressure created by unrestricted use of antimicrobial drugs in human medicine, veterinary medicine, and aquaculture. Development of a countrywide antimicrobial resistance surveillance system in livestock, wildlife species, and humans in Bangladesh, as well as other measures, are needed immediately to control the situation.
